# Improving Physical Health Assessments in Alcohol Use Disorder: A Service Audit

**DOI:** 10.1192/bjo.2025.10627

**Published:** 2025-06-20

**Authors:** Mario Lepore, Chris Joseph, Mike Kelleher

**Affiliations:** South London and Maudsley NHS Foundation Trust, London, United Kingdom

## Abstract

**Aims:** Individuals seeking support from drug and alcohol services often experience significant physical health challenges, with many presenting with comorbid conditions such as liver disease. Early identification of these conditions is vital for ensuring holistic and effective patient care. This audit aimed to evaluate the proportion of clients with alcohol use disorders who had recently undergone blood tests, Fibrosis-4 (FIB-4) scoring (a non-invasive marker for liver fibrosis) and FibroScan. The audit also sought to identify gaps in current clinical practices and provide recommendations to optimise care pathways for this population.

**Methods:** The medical records of 200 clients, representing two-thirds of the total population with alcohol as their primary substance of use, were reviewed. Data were collected to determine whether clients had received necessary blood tests (including liver function and platelet count) to facilitate FIB-4 scoring, and whether FibroScan assessments had been offered or completed. Reasons for incomplete investigations were recorded to identify potential barriers to care.

**Results:** Out of the 200 clients, 175 (87.5%) had recent blood test results and 134 (67%) had all components required to calculate FIB-4 scores. A total of 90 (40%) had undergone Fibroscan testing. Several factors contributed to incomplete investigations, including client refusal or non-engagement, incomplete blood test requests and failure to reoffer FibroScans to those with abnormal liver function tests. With regards to incomplete blood tests, it was identified that aspartate aminotransferase (AST) levels were not routinely measured unless a specific request for this was made, something not all staff members were aware of.

**Conclusion:** The audit highlighted factors contributing to gaps in the completion of necessary blood tests and FibroScans among individuals attending alcohol treatment services. In response, we have implemented targeted clinician training, updated blood test request protocols to ensure all necessary components for FIB-4 calculation are included, and revised guidelines to ensure FibroScan is reoffered to clients with abnormal liver function tests. These improvements are expected to enhance the consistency and quality of physical health assessments for this vulnerable group. A follow-up audit will be conducted in six months to assess progress.

